# Women are lean and men are also lean: nutrition titles in women’s and men’s health magazines

**DOI:** 10.1186/s12889-024-18706-4

**Published:** 2024-05-03

**Authors:** Hélène Burdet, Aris Xanthos, Pedro Marques-Vidal

**Affiliations:** 1 Faculty of Biology and Medicine, University of Lausanne, Lausanne, Switzerland; 2 Department of Language and Information Science, University of Lausanne, Bâtiment Anthropole, Lausanne, 1015 Switzerland; 3 Department of medicine, internal medicine, Lausanne university hospital and University of Lausanne, 46 rue du Bugnon, Lausanne, 1011 Switzerland

**Keywords:** Nutrition, Weight gain, Weight loss, Lay media, Gender differences

## Abstract

**Background:**

Whether nutrition messages in popular health magazines differ by country or season has seldom been studied. We assessed the nutrition topics featured in the headlines of Men’s Health® (MH) and Women’s Health® (WH) magazines from different countries.

**Methods:**

We sampled MH and WH magazines from Portugal, South Africa, Spain, the UK and the USA. Nutrition-related headlines were categorized as weight loss, weight gain, micronutrients and other.

**Results:**

The most frequent topics were “Other” (44%) and “weight loss” (41%), while “micronutrients” represented 4%. Topics related to weight gain were more frequent in MH (19% vs. 2% in WH), while no difference was found for weight loss (44% vs. 37% in WH). On multivariable analysis, weight gain had a higher likelihood of being present in MH than in WH, Odds ratio and (95% confidence interval): 8.3 (2.2–90.9), *p* = 0.002, while no association was found for weight loss: OR 1.1 (0.6-2.0), *p* = 0.80. Weight loss was absent from the US WH and present in two thirds of the Portuguese WH; in MH, weight gain was evenly distributed between countries. Prevalence of the weight loss topic was lower in March (15% vs. 54% in January, *p* < 0.01 by logistic regression) and to a lesser degree in June (35%) and July (35%). No seasonality was found for the “weight gain” topic.

**Conclusion:**

In WH and MH magazines, nutrition topics vary according to gender, country, and season. Weight gain remains a male topic, while weight loss is equally prevalent in both women’s and men’s magazines.

**Supplementary Information:**

The online version contains supplementary material available at 10.1186/s12889-024-18706-4.

## Introduction

Unhealthy diets such as diets rich in sodium or fat, or poor in fruits and vegetables, were responsible for 11 million deaths (22% of all deaths) among adults in 2017 [1]. Unhealthy diets increase the risk of dying from a noncommunicable disease [2] and of developing obesity. Indeed, according to the World Health Organization, obesity rates have tripled between 1975 and 2016, over 650 million adults presenting with obesity and 1.9 billion with overweight in 2016 [3].

More than a health issue, nutrition is also a valuable topic for the media. Nutrition is prominently featured in advertising, in health magazines and on social media. Nutrition is associated with images of an ideal body, an optimal weight, or a healthy diet. Media play a significant role in shaping beauty ideals, showing what an attractive body should look like for women: a lean, beautiful, practically unattainable body, often causing body dissatisfaction [4]. The same applies to men, young, muscular, athletic bodies being promoted in most if not all health magazine covers [5]. Further, there is an overabundance of nutritional information in the media, contradictory messages being commonplace [6]. Indeed, many articles in the media either overvalue the effects of “superfoods” or “miracle foods” with no solid scientific evidence or promise amazing but impossible results [7]. The topics covered also tend to follow a seasonal pattern, articles focusing on weight loss being more frequent in the summer or post-New Year [8, 9], possibly leading to weight cycling or seasonal body dissatisfaction [9].

Some magazines focusing on health reach very large audiences. For instance, Women’s Health® magazine claims over 8 million readers worldwide [10], while Men’s Health® magazine claims 9 million readers [11]. A study focused on Men’s Health® UK magazine reported that nutrition advice was mostly oriented towards muscularity and leanness, and less oriented towards body weight control. Interestingly, many scientific sources were cited to endorse the claims and dietary advice. Still, the authors concluded “Despite the widespread use of scientific information to endorse dietary advice, the content, format and scientific basis of dietary content of MH leaves much to be desired. The dietary advice as provided may not be conducive to public health.” [12].

Most studies on nutritional messages provided by the lay media focused on a single country or a single magazine. Whether the nutritional topics covered by a given magazine are similar irrespective of the country of publication is something that has not been assessed. This knowledge gap is important to fill, as nutrition recommendations for the general population are quite similar irrespective of the country [13]. To our knowledge, no study assessed nutritional claims in magazines from different countries and compared magazines aimed at a male or female readership. As health and body objectives might change according to gender [14], country [15], and season [9], we aimed to evaluate nutrition statements in the headlines of Men’s Health® and Women’s Health® magazines from several countries for a 3-year period. Our hypothesis was that the topics related to weight loss would be more prevalent in the women’s magazines, while topics related to muscle and weight gain would be more prevalent in the men’s magazines. We also hypothesized that headlines related to weight loss or weight gain would follow a seasonal pattern.

## Methods

### Magazines

We sampled Men’s Health® (MH) and Women’s Health® (WH) magazines from five countries (Portugal, South Africa, Spain, the UK, and the USA). The sampling included all issues available at the Library of the University of Lausanne. The number of issues collected, and year range is indicated in supplementary Table 1. The magazines are published monthly.

### Titles / headlines extraction

Titles that could be related to nutrition were extracted and further coded into four categories: weight loss, weight gain, micronutrients and other. Weight loss and weight gain were selected based on previous studies [8, 16, 17] and on the importance of weight loss for the media [18] and the general population [19, 20]. The same rationale was applied for micronutrients [12]. The “other” category included all other topics, for examples recipes “one-dish dinner ideas” (WH South Africa); foods “Spicy! Foods that bring the healthy heat” (WH USA), diets “Es segura la keto?” (MH Spain), eating out “The UK’s 29 healthiest restaurants. Eat out without blowing out” (MH UK) or miscellaneous “What do nutrition experts really say?” (WH UK). As titles focusing on weight loss or weight gain could be related with exercise and not with nutrition, the content of the related article was scanned if necessary. When neither nutrition nor exercise were indicated to gain or to lose weight, for example “Lose 4 kg in one month” (MH Portugal), the title was still included in the analysis. The coding was conducted by the first author (HB) and checked by a senior investigator (PMV).

### Statistical analysis

Statistical analysis was conducted focusing on the seasonality of topics and their distribution according to target gender or country. Comparison of topics between gender (i.e., MH vs. WH) and countries stratified by gender was performed by chi-square or Fisher’s exact test. Seasonality was assessed by comparing the frequency of occurrence of one topic from February to December relative to the frequency observed in January. This method was selected as most weight loss / weight gain decisions are made at the beginning of the year. The comparison was performed using logistic regression and the results were expressed as odds ratio and (95% confidence interval). Statistical analyses were performed using Stata version 16.1 (Stata Corp, college station, TX, USA) and statistical significance was considered for a two-sided test with *p* < 0.05.

## Results

The number of issues analysed by target gender and country are summarized in supplementary Table 1. Overall, 301 issues were analysed, 138 from WH and 163 from MH.

**Fig. 1:**
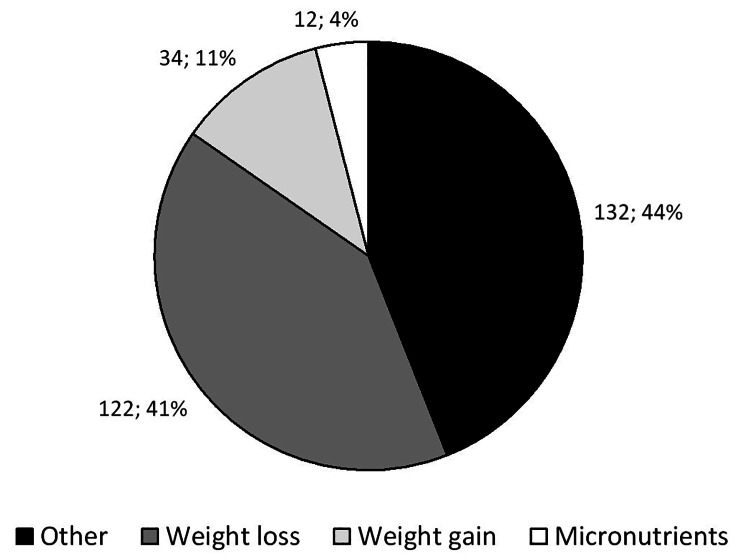
distribution of nutrition-related topics in the headlines of Women’s Health® and Men’s Health® magazines, 2018–2022. Results are provided as number of topics and overall percentage

Overall, the main topics presented in the headlines were “other”, followed by “weight loss”, “weight gain” and “micronutrients” (Fig. 1). Topics were unevenly distributed according to readership, “other” being more frequent in WH, and “weight gain” and “weigh loss” more frequent in MH (Table 1).

**Table 1:** topics in the headlines of Women’s Health® and Men’s Health® magazines, 2018–2022

Topic, *n* (%)	Women’s Health®	Men’s Health®	*p*-value
Sample size	137	163	< 0.001
Other	79 (57.7)	53 (32.5)	
Weight loss	50 (36.5)	72 (44.2)	
Weight gain	3 (2.2)	31 (19.0)	
Micronutrients	5 (3.7)	7 (4.3)	

Results are expressed as number and (column percentage). Statistical analysis of the distribution of topics between magazines performed by chi-square

The distribution of the topics according to country, overall and stratified by gender, is summarized in Table 2. Overall, topics were unevenly distributed between countries; when the analysis was stratified by readership, the distribution differed between WH magazines, Portugal presenting a higher frequency of “weight loss” and South Africa and the USA a majority of “other”. No differences in the distribution of topics were found between countries in MH magazines.

**Table 2:** topics in the headlines of Women’s Health® and Men’s Health® magazines, 2018–2022, according to country

Topic, *n* (%)	Portugal	South Africa	Spain	UK	USA	*p*-value
**Overall, sample size**	91	50	29	78	52	
Micronutrients	4 (4.4)	2 (4.0)	1 (3.5)	4 (5.1)	1 (1.9)	< 0.001
Other	22 (24.2)	33 (66.0)	10 (34.5)	32 (41.0)	35 (67.3)	
Weight gain	15 (16.5)	0 (0)	5 (17.2)	8 (10.3)	6 (11.5)	
Weight loss	50 (55.0)	15 (30)0.0	13 (44.8)	34 (43.6)	10 (19.2)	
**Women, sample size**	**29**	**50**		**36**	**22**	
Micronutrients	1 (3.5)	2 (4.0)	-	2 (5.6)	0 (0)	< 0.001§
Other	9 (31.0)	33 (66.0)	-	15 (41.7)	22 (100)	
Weight gain	0 (0)	0 (0)	-	3 (8.3)	0 (0)	
Weight loss	19 (65.5)	15 (30.0)	-	16 (44.4)	0 (0)	
**Men, sample size**	**62**		**29**	**42**	**30**	
Micronutrients	3 (4.8)	-	1 (3.5)	2 (4.8)	1 (3.3)	0.473§
Other	13 (21.0)	-	10 (34.5)	17 (40.5)	13 (43.3)	
Weight gain	15 (24.2)	-	5 (17.2)	5 (11.9)	6 (20.0)	
Weight loss	31 (50.0)	-	13 (44.8)	18 (42.9)	10 (33.3)	

-, no data. Results are expressed as number (column percentage) Statistical analysis of the distribution of topics between countries performed by chi-square or Fisher’s exact test (§)

**Fig. 2:**
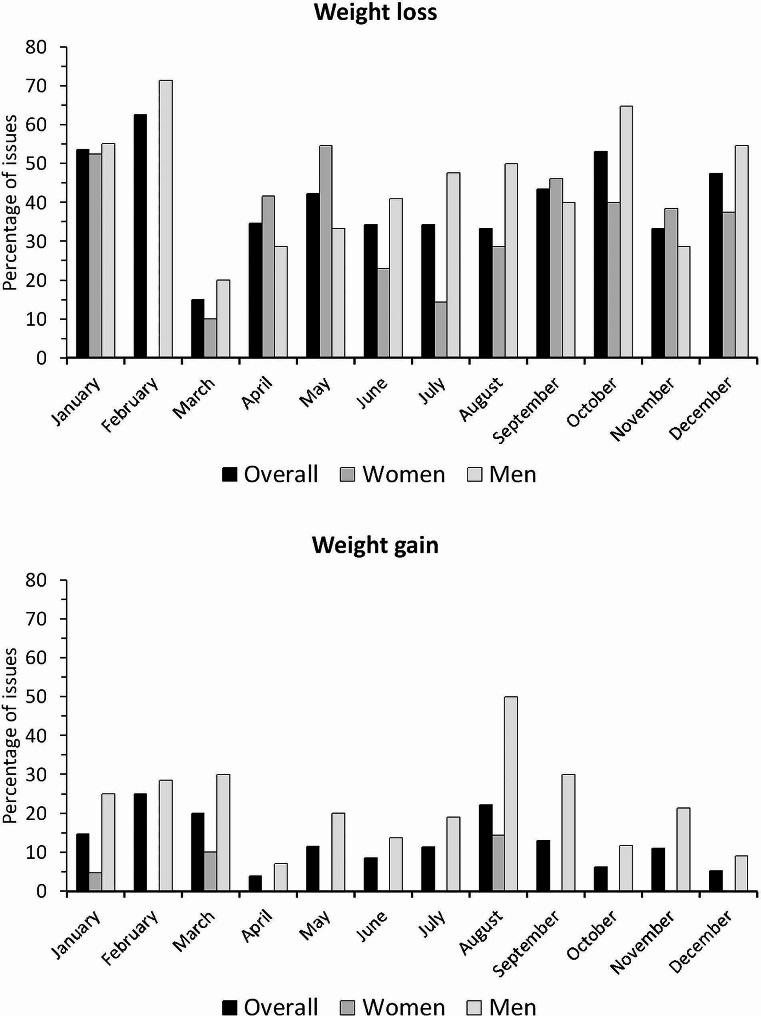
seasonality of topics related to weight loss (upper panel) and weight gain (lower panel) in the headlines of Women’s Health® and Men’s Health® magazines, 2018–2022

The seasonality of the topics regarding weight loss and weight gain, overall and stratified by gender, is summarized in Fig. 2. Weight loss was more frequent in January and May for WH and in February and October for MH, and less frequent in March irrespective of the readership. Weight gain was more frequent in August in MH, but the differences were not statistically significant. As season is different in the Southern hemisphere, an analysis on weight loss in WH magazines after excluding the issues from South Africa was conducted; a similar pattern was found, with a nadir occurring in February-March and peak occurring in May (supplementary Fig. 1).

When logistic regression was applied, March and July were less likely to present topics related to weight loss overall and in WH, while no clear seasonality was found for weight gain (Table 3). Similar results were found after excluding South Africa (supplementary Table 2).

**Table 3:** seasonality of topics related to weight gain or weight loss in the headlines of Women’s Health® and Men’s Health® magazines, 2018–2022

			Weight loss						Weight gain	
Month	Overall	*p*-value	Women	*p*-value	Men	*p*-value	Overall	*p*-value	Men	*p*-value
January	1 (ref.)		1 (ref.)		1 (ref.)		1 (ref.)		1 (ref.)	
February	1.44 (0.30–6.83)	0.647	-		2.05 (0.32–13.2)	0.451	1.94 (0.32-12.0)	0.474	1.20 (0.17–8.24)	0.853
March	0.15 (0.04–0.60)	0.007	0.10 (0.01–0.95)	0.045	0.20 (0.03–1.22)	0.081	1.46 (0.36–5.89)	0.596	1.29 (0.24–6.96)	0.771
April	0.46 (0.17–1.26)	0.131	0.65 (0.16–2.72)	0.555	0.33 (0.08–1.40)	0.133	0.23 (0.03–2.06)	0.190	0.23 (0.02–2.24)	0.206
May	0.63 (0.24–1.71)	0.366	1.09 (0.25–4.71)	0.907	0.41 (0.10–1.64)	0.207	0.76 (0.17–3.35)	0.718	0.75 (0.15–3.79)	0.728
June	0.45 (0.18–1.14)	0.093	0.27 (0.06–1.28)	0.100	0.57 (0.17–1.93)	0.363	0.55 (0.13–2.37)	0.420	0.47 (0.10–2.31)	0.355
July	0.45 (0.18–1.14)	0.093	0.15 (0.03–0.85)	0.032	0.74 (0.22–2.54)	0.637	0.75 (0.19–2.92)	0.681	0.71 (0.16–3.12)	0.646
August	0.43 (0.09–1.97)	0.278	0.36 (0.06–2.31)	0.284	0.82 (0.04-15.0)	0.892	1.67 (0.28-10.0)	0.577	3.00 (0.16–57.4)	0.466
September	0.66 (0.24–1.86)	0.435	0.78 (0.19–3.12)	0.724	0.55 (0.12–2.55)	0.441	0.88 (0.20–3.89)	0.861	1.29 (0.24–6.96)	0.771
October	0.98 (0.39–2.47)	0.964	0.61 (0.16–2.32)	0.464	1.50 (0.40–5.66)	0.550	0.39 (0.07–2.07)	0.269	0.40 (0.07–2.39)	0.316
November	0.43 (0.16–1.18)	0.103	0.57 (0.14–2.32)	0.431	0.33 (0.08–1.40)	0.133	0.73 (0.17–3.20)	0.676	0.82 (0.16–4.17)	0.809
December	0.78 (0.26–2.31)	0.650	0.55 (0.10–2.89)	0.476	0.98 (0.22–4.30)	0.981	0.32 (0.04–2.90)	0.314	0.30 (0.03–2.97)	0.303

-, no data. Results are expressed as odds ratio (95% confidence interval) Statistical analysis by logistic regression. For weight gain, the paucity of data in women’s magazines precluded the analysis

## Discussion

Our results show that the nutrition topics presented in the headlines of WH and MH magazines vary considerably between genders, countries and to a lesser degree by season.

### Main topics in headlines

Besides “other” nutritional topics, “weight loss” ranked high in the topics list and was almost as prevalent in WH and in MH magazines, with the notable exception of WH from the USA. Obesity affects four out of ten Americans [21], and 55% of them want to lose weight [22]; still, no headline related to weight loss was found in WH of the USA. The reason might be editorial, due either to the impact of the fat acceptance movement [23], or to the fear of promoting fat shaming [24]. Yet, the women depicted in the covers of WH USA had a lean body, suggesting that WH USA might not be this body inclusive. Interestingly, the high prevalence of “weight loss”-related headlines in MH magazines was unexpected, and outnumbered the “weight gain” topic, which is traditionally a male’s concern. The prevalence of the “weight loss” headlines was almost similar between WH and MH magazines from Portugal and the UK, while in the USA the “weight loss” topic was only shown in MH. Again, the reason might be editorial, aimed at increasing readership by people with overweight or obesity. Another possibility would be an increase in body dissatisfaction and for thinness among men, although this statement has been challenged [25]. Overall, our results indicate that weight loss is no longer only a woman-related topic, being now more frequent among men’s magazines.

Weight gain was almost exclusively a MH topic. Weight gain in MH was mostly related to increased muscle, a finding also reported previously [12]. Indeed, most MH covers featured muscular, popular sports or movie stars, a strategy also reported elsewhere [5, 26]. Hence, magazines might use both the “weight loss” and the “weight gain” topics to motivate/convince readers that they can change their bodily features from fat to muscular [27]. Conversely, such strategies might also lead to increased body dissatisfaction [28], increased demand for quick results, leading to weight loss strategies that are more deleterious than beneficial [7].

Overall, our results indicate that the topics covered in the headlines of WH and MH differ according to target readership and country. The differences observed by country could be due to the willingness of the editorial boards to target specific parts of the general population to ensure a good acceptance of the magazine. Studies on the characteristics of the readers within each country are needed to better assess this point.

### Seasonality

In both WH and MH, “weight loss” headlines were more frequent in January, coinciding with New Year’s resolutions. Indeed, losing weight is one of the most frequent New Year’s resolutions [29, 30], and our findings agree with a previous study reporting that many weight loss messages are published at the beginning of the year [8]. In WH, there were almost no headlines related to weight loss in February and March, the topic gaining again prominence before summer. The probable explanation is to become “beach ready” and avoid the apprehension of summer body dissatisfaction [31]. The number of headlines related to weight loss then decreased during summer, to rise again at the end of the year. This rebound also appeared in MH and might be aimed at counterbalancing the weight gain of the Christmas season [17]. Unlike in WH, in MH there was no increase in the number of headlines related to weight loss before summer. This might be because the pressure to be thin is higher for women than it is for men. Regarding the headlines related to weight gain, the greater frequency occurred in August, which does not seem to be aimed at being “beach ready” as it occurs too late. A possible explanation is that it is aimed at pushing the readers to become more muscular, as it was shown that men internalize the media’s drive for muscularity [32, 33].

### Implications for public health

Health is an important public topic, and health magazines can exert a powerful impact in the general population via the messages they convey. The importance of the weight loss and weight gain topics found in WH and MH magazines carries the image of an idealized, thin, and healthy self. This image might be unattainable by many readers, leading to body dissatisfaction and eventually disordered eating behaviors [4, 34]. Also, the seasonality of the headlines related to weight loss could induce weight cycling, with possible deleterious effects in the cardiovascular system [35]. This seasonality could also impact body image among readers, increasing their body dissatisfaction during Summer [9]. Hence, in presence of disordered eating or body image issues, health professionals should search for the consultation of health magazines and eventually prevent their reading by the patients.

### Strengths and limitations

To our knowledge, this is the largest study assessing nutrition topics on health magazines, and the only one that compared the same magazine published in different countries. Indeed, previous studies focused on a magazine from a single country [12]. Other studies provided trends in nutrition but did not assess seasonality [36].

This study has also some limitations worth acknowledging. First, it selected magazines in languages accessible to the authors; hence, magazines from other countries could not be sampled and generalizability of the findings might not be achievable. Secondly, due to the different languages of the magazines, it was not possible to analyse the tones, the words used and their synonyms, or the rhetoric of the headlines. More in-depth textual analysis will be necessary. Thirdly, only the headlines in the cover pages were collected and analysed, not the full length of the articles. However, the titles are most likely a good representation of the content of the articles, as the role of a title is to convey the subject covered. Finally, in many cases the headlines nether nutrition nor exercise were indicated as means to lose or gain weight. Hence, it was not possible to establish if the headline focused on nutrition, on exercise, or both.

## Conclusion

In WH and MH magazines, nutrition topics vary according to gender, country, and season. This variation could increase body dissatisfaction or lead to inadequate eating behaviours among vulnerable individuals. Weight gain remains a male topic, while weight loss is equally prevalent in both women’s and men’s magazines.

### Electronic supplementary material

Below is the link to the electronic supplementary material.

Supplementary Material 1

## Data Availability

Data can be provided upon request. Please contact Prof. Pedro Marques-Vidal (Pedro-Manuel.Marques-Vidal@chuv.ch).
