# Supplementary material for: Women are lean and men are also lean: nutrition titles in women’s and men’s health magazines
Source: BMC Public Health. 2024 May 3;24:1230. doi: 10.1186/s12889-024-18706-4 (PMC11069188; doi:10.1186/s12889-024-18706-4)

**Supplementary information**

**Supplementary table 1**: number of issues, years and countries from the magazines sampled.

| **Magazine name** | **Country** | **N of issues** | **Year range** | **Number of readers *** |
| --- | --- | --- | --- | --- |
| Women’s health | Portugal | 29 | 2018-2022 |  |
| Women’s health | South Africa | 51 | 2018-2020 |  |
| Women’s health | UK | 36 | 2019-2022 | 893,000 ^a^ |
| Women’s health | USA | 22 | 2019-2022 | 150,0000 ^b^ |
| Men’s health | Portugal | 62 | 2019-2022 |  |
| Men’s health | Spain | 29 | 2019-2022 |  |
| Men’s health | UK | 42 | 2019-2022 | 1,800,000 ^c^ |
| Men’s health | USA | 30 | 2019-2022 |  |

* if available.

^a^ <https://www.womenshealthmag.com/uk/health/a25002958/about-womens-health-uk/>

^b^ <https://en.wikipedia.org/wiki/Women%27s_Health_(magazine)>

^c^ <https://www.statista.com/statistics/381735/men-s-health-monthly-reach-by-demographic-uk/#:~:text=Men%27s%20Health%20magazine%20had%20an,its%20website%20during%20this%20period>.

**Supplementary table 2**: seasonality of topics related to weight loss in the headlines of Women’s Health® and Men’s Health® magazines, 2018-2022, overall and stratified by readership, excluding South Africa.

| **Month** | **Overall** | **p-value** | **Women** | **p-value** |
| --- | --- | --- | --- | --- |
| January | 1 (ref.) |  | 1 (ref.) |  |
| February | 1.48 (0.30 - 7.21) | 0.626 | - |  |
| March | 0.13 (0.02 - 0.65) | 0.013 | - |  |
| April | 0.41 (0.14 - 1.27) | 0.124 | 0.60 (0.10 - 3.54) | 0.572 |
| May | 0.74 (0.25 - 2.17) | 0.585 | 2.50 (0.36 - 17.5) | 0.356 |
| June | 0.52 (0.19 - 1.47) | 0.218 | 0.25 (0.02 - 2.84) | 0.263 |
| July | 0.59 (0.22 - 1.60) | 0.302 | 0.29 (0.04 - 1.89) | 0.194 |
| August | 0.59 (0.09 - 4.01) | 0.592 | 0.50 (0.04 - 6.86) | 0.604 |
| September | 0.73 (0.24 - 2.20) | 0.574 | 1.00 (0.20 - 5.07) | 1.000 |
| October | 1.26 (0.46 - 3.42) | 0.651 | 1.00 (0.21 - 4.67) | 1.000 |
| November | 0.41 (0.14 - 1.27) | 0.124 | 0.60 (0.10 - 3.54) | 0.572 |
| December | 1.02 (0.30 - 3.43) | 0.980 | 1.00 (0.11 - 9.23) | 1.000 |

-, topic absent from all issues. Results are expressed as odds ratio (95% confidence interval). Statistical analysis by logistic regression.

**Supplementary figure 1:** seasonality of topics related to weight loss in the headlines of Women’s Health® magazines, 2018-2022, with or without South Africa.


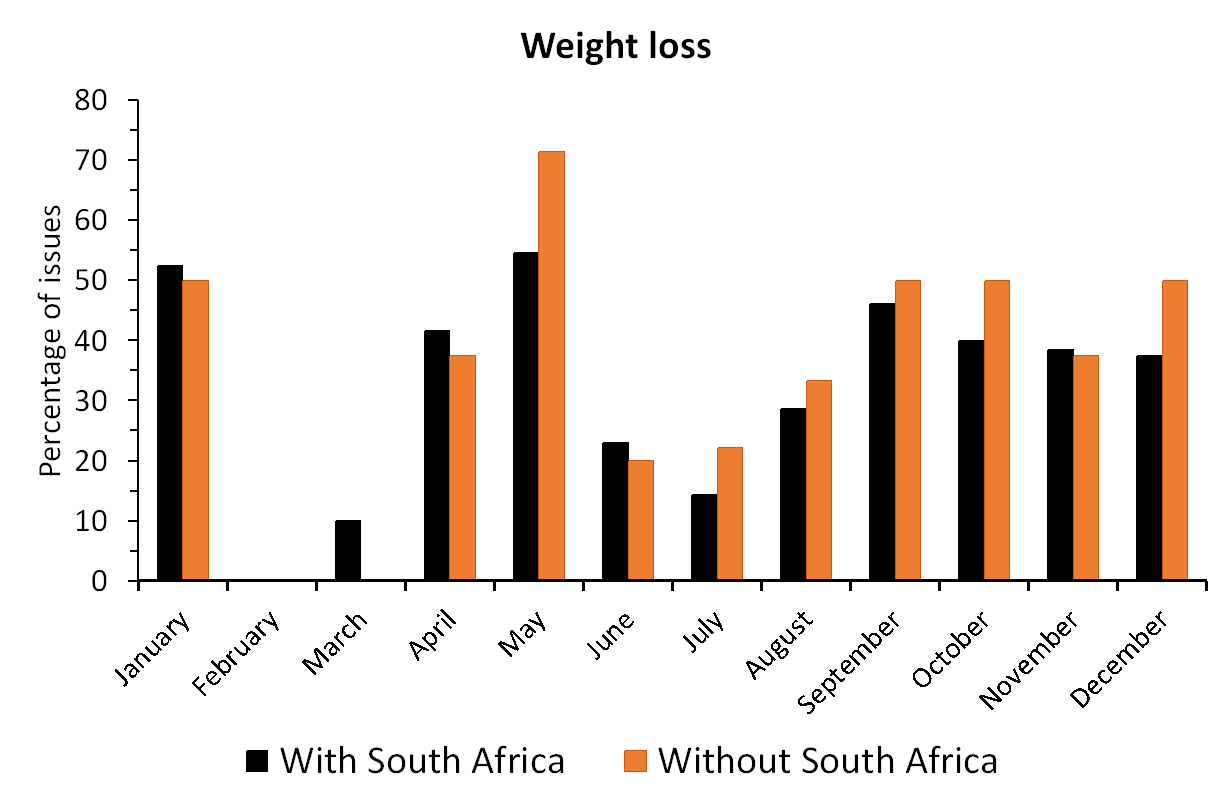

Supplement: Supplementary file 1 — Supplementary Material 1 [file 12889_2024_18706_MOESM1_ESM.docx]
